# Supplementary material for: A prospective randomized trial on abacavir/lamivudine plus darunavir/ritonavir or raltegravir in HIV-positive drug-naïve patients with CD4<200 cells/uL (the PRADAR study)
Source: PLoS One. 2019 Sep 27;14(9):e0222650. doi: 10.1371/journal.pone.0222650 (PMC6764686; doi:10.1371/journal.pone.0222650)
Supplement: S2 File — Original Protocol. (DOC) [file pone.0222650.s002.doc]

**A prospective, randomized, open-label trial of two abacavir/lamivudine based regimen (ABC/3TC + darunavir/ritonavir or ABC/3TC + raltegravir) in late presenter naïve patients (with CD4 count <200 cells/µL - advanced HIV disease).**

Study No. RTLP

Version No: 1.2

Date: 23 February 2012

Principal Investigator: Prof.ssa Cristina Mussini

Infectious Diseases Clinic
University Hospital - Policlinico
Via del Pozzo, 71
41124 Modena, Italy

Phone: 0039 059 4222466

e mail: cristina.mussini@unimore.it

**T**ABLE OF CONTENTS

[1. PROTOCOL SUMMARY 3](#__RefHeading___Toc317843275)

[1.1 Clinical Objectives: 3](#__RefHeading___Toc317843276)

[1.2 Study population: 3](#__RefHeading___Toc317843277)

[1.3 Outcome 3](#__RefHeading___Toc317843278)

[1.4 Study design 4](#__RefHeading___Toc317843279)

[1.6 Treatment regimens: 4](#__RefHeading___Toc317843280)

[1.7 Criteria for Safety: 4](#__RefHeading___Toc317843281)

[1.8 Statistical analysis: 4](#__RefHeading___Toc317843282)

[2. INTRODUCTION 5](#__RefHeading___Toc317843283)

[2.1 Background 5](#__RefHeading___Toc317843284)

[2.2 Rationale 5](#__RefHeading___Toc317843285)

[3. STUDY DESIGN 7](#__RefHeading___Toc317843286)

[4. OBJECTIVES 8](#__RefHeading___Toc317843287)

[5. OUTCOMES 8](#__RefHeading___Toc317843288)

[6. TARGET POPULATION 8](#__RefHeading___Toc317843289)

[6.1 Inclusion criteria 8](#__RefHeading___Toc317843290)

[6.2 Exclusion Criteria 9](#__RefHeading___Toc317843291)

[6.3 Number of subjects 9](#__RefHeading___Toc317843292)

[6.4 Removal of subjects from study 9](#__RefHeading___Toc317843293)

[7. TREATMENT REGIMENS 10](#__RefHeading___Toc317843294)

[7.1 Prior and concomitant therapy 10](#__RefHeading___Toc317843295)

[7.2 Discontinuation 10](#__RefHeading___Toc317843296)

[8. VISIT SCHEDULE 11](#__RefHeading___Toc317843297)

[8.1 Physical examination 11](#__RefHeading___Toc317843298)

[8.2 Clinical Laboratory Tests 11](#__RefHeading___Toc317843299)

[8.3 Pregnancy test 12](#__RefHeading___Toc317843300)

[8.4 Data collection 12](#__RefHeading___Toc317843301)

[8.5 Adherence and Neurocognitive Evaluation 12](#__RefHeading___Toc317843302)

[9. STATISTICAL ANALYSIS 12](#__RefHeading___Toc317843303)

[10. ADVERSE EVENTS (AEs) AND SERIOUS ADVERSE EVENTS (SAEs) 13](#__RefHeading___Toc317843304)

[10.1 Definition of an adverse event (AE) 13](#__RefHeading___Toc317843305)

[10.2 Assessment of Adverse Event Severity and Relationship to Treatment 14](#__RefHeading___Toc317843306)

[10.3 Definition of a serious adverse event (SAE) 14](#__RefHeading___Toc317843307)

[10.4 Abnormal laboratory parameters and other abnormal values as AEs or SAEs 15](#__RefHeading___Toc317843308)

[10.5 Reporting AEs and SAEs 15](#__RefHeading___Toc317843309)

[10.8 Deadlines for SAE notification 16](#__RefHeading___Toc317843310)

[10.9 Suspected Unexpected Serious Adverse Reactions (SUSARs) 16](#__RefHeading___Toc317843311)

[11. ETHICAL and REGULATORY ASPECT 16](#__RefHeading___Toc317843312)

[11.1 Regulatory Authority Approval 16](#__RefHeading___Toc317843313)

[11.2 Ethical Approval 16](#__RefHeading___Toc317843314)

[11.3 Informed Consent Form 17](#__RefHeading___Toc317843315)

[11.4 Duties of Investigator 17](#__RefHeading___Toc317843316)

[11.5 Trial Documents and Records Retention 17](#__RefHeading___Toc317843317)

[11.6 Monitoring Arrangements 18](#__RefHeading___Toc317843318)

[12. PUBBLICATION 18](#__RefHeading___Toc317843319)

[Appendix 1:VAS scale for adherence 19](#__RefHeading___Toc317843322)

[Appendix 2: Neurocognitive evaluation 20](#__RefHeading___Toc317843323)

[Flowchart 24](#__RefHeading___Toc317843324)

[II. Definitions of terms used in the Table: 26](#__RefHeading___Toc317843325)

[References 42](#__RefHeading___Toc317843326)

# 1. PROTOCOL SUMMARY

This is a prospective, randomized open-label, 2 arm, 3-phase trial to compare the 48-weeks virological response of two different regimens containing abacavir/lamivudine (abacavir/lamivudine +darunavir/ritonavir (DRV/r) vs abacavir/lamivudine + raltegravir (RAL) in antiretroviral therapy naive, HIV+ individuals presenting for care with CD4+ counts < 200/mm^3^.

## 1.1 Clinical Objectives:

**Primary Objective:** To compare the 48-week virological response to two different regimens containing abacavir/lamivudine (abacavir/lamivudine +darunavir/ritonavir (DRV/r) vs abacavir/lamivudine + raltegravir (RAL) in antiretroviral therapy naive, HIV+ individuals presenting for care with CD4+ counts < 200/mm^3^.

**Secondary Objective:** a) To compare immunological response at 48 weeks;

b) To determine the safety and tolerability of the 2 regimens.

1.2 Study population: 350 inpatients or outpatients will be randomized

## 1.3 Outcome

**Primary Endpoint**

- Proportion of patients with undetectable viremia (HIV RNA<50 copies/mL) after 48 weeks

**Secondary Endpoints(s)**

- Change in CD4+ cell count from baseline through week 48
- Time to virological rebound

**Safety endpoints**

- Incidence of adverse events (AEs)
- Incidence of serious adverse events (SAEs)
- Discontinuations due to adverse events
- Incidence of grade 3 or 4 laboratory abnormalities.

## 1.4 Study design

Multicentre, parallel group, randomised, open label, non-inferiority study

**1.5** Planned sample size**:** The planned sample size for this trial is 350 patients

## 1.6 Treatment regimens:

Arm A: abacavir/lamivudine 1 tablet once a day + raltegravir 400 mg (1 tablet twice a day)

Arm B: abacavir/lamivudine 1 tablet once a day + ritonavir 100 mg + darunavir 800 mg once a day.

All drugs have been approved for the treatment of HIV infection.

Administration: oral

The study population will consist of 350 HIV-positive, HLA B5701-negative patients. At baseline, patients will be randomized 1:1 to start abacavir/lamivudine plus either raltegravir or darunavir/ritonavir. Randomization will be stratified on the basis of the screening CD4+ cell count (≤100 vs ≥100 cells/µL), to ensure balance across treatments groups

1.7 Criteria for Safety: Adverse events and laboratory assessments.

1.8 Statistical analysis:

As this is a non-inferiority trial, we will calculate the difference in the proportions of patients experiencing the primary outcome in the two treatment arms and will calculate a 95% confidence interval for this.  Non-inferiority of the raltegravir arm will be demonstrated if the lower limit of the 95% confidence interval is greater than -12%. In case non-inferiority will be met, analyses for superiority will be performed.

# 2. INTRODUCTION

## 2.1 Background

Advanced HIV disease, defined as a CD4 cell count <200 cells/µL or the presence of an AIDS-defining event, remains common among HIV-infected individuals who first present for medical care either in developing or in developed countries. Moreover, as shown by a paper published from our group, the percentage of patients presenting with these characteristics has remained relatively stable over the last 15 years *[1]*.

These patients represent a challenge for clinicians; not only do they frequently present with one or more opportunistic infections, but treatment strategies should also take into account the possible symptomatic or asymptomatic involvement of central nervous system (CNS). To date, the only trial that has addressed the question of “what to start” in this population is a Mexican study [2] who reproduced the ACTG 5142 trial comparing efavirenz with lopinavir, with combivir as the nucleoside backbone [3]. Results were similar to those from ACTG 5142, showing a superiority of efavirenz for the virological end-point, but a greater increase in CD4 cells among patients randomised to lopinavir. Despite the fact that this was the first trial conducted in a population of patients with advanced HIV infection, the results may already be perceived to be somewhat out-of-date, as neither lopinavir or combivir are considered as preferred choices for first-line therapy in many Western countries.

## 2.2 Rationale

We believe that abacavir/lamivudine, together with either raltegravir or darunavir/ritonavir (800/100), are suitable combinations for use in patients with advanced HIV infection. Although tenofovir is a preferred component of the NRTI backbone in most international treatment guidelines, we do not feel that its use is recommended in this population for several reasons: (i) it has almost no CNS penetration; (ii) patients with advanced HIV infection are likely to be receiving other drugs for opportunistic infections, many of which are associated with renal toxicity; and (iii) a high proportion of the population already have osteopenia/osteoporosis [4]. We believe that abacavir/lamivudine would be preferable in this population due to its tolerability, potency and high CNS penetration score. Whilst there are concerns about the development of hypersensitivity in those receiving abacavir, all patients are now screened for HLA B5701 (or at least HLA B57) and so this side effect is now infrequent. Concerning potency, ACTG 5202 is, to date, the only randomized trial that has formally compared abacavir/lamivudine to tenofovir/emtricitabine. A key finding from this study was that in the stratum of patients with plasma HIV RNA >100000 copies/mL, virological responses were better in patients receiving tenofovir/emtricitabine [5].

Nevertheless, we feel confident that we will be able to use abacavir/lamivudine as a backbone in patients with advanced HIV infection, many of whom will have a high plasma HIV RNA level, for several reasons:

- - 1. In ACTG 5202, patients were not tested for HLA B5701. As the analysis was performed on an intent-to-treat basis, a large part of the effect seen may have been explained by differences in tolerability between the arms.

- - 1. Only a minority of subjects in ACTG 5202 underwent genotypic resistance testing at baseline. Since abacavir/lamivudine has a lower genetic barrier than tenofovir/emtricitabine, virological responses may have been detrimentally affected, particularly in the efavirenz arm.
    2. ACTG 5202 considered two different third drugs: efavirenz and atazanavir/ritonavir. Given the rapid decline in HIV RNA seen in patients receiving raltegravir, this drug may be a more potent companion for abacavir/lamivudine than efavirenz [6]. Equally, the higher genetic barrier of darunavir/ritonavir than atazanavir/ritonavir may mean that this drug may also be preferable.
    3. A previous study conducted by Smith et al reported equivalence between abacavir/lamivudine and tenofovir/emtricitabine when combined with lopinavir/ritonavir [7].
    4. An analysis of the UK CHIC Study also showed no difference in virological outcomes between patients receiving either abacavir or tenofovir in combinations with lamivudine or emtricitabine [8].
    5. To exclude patients at particularly high risk of virological failure, we will exclude patients from this trial if they have an HIV RNA level >500,000 copies/mL.

The choice of darunavir/ritonavir (or raltegravir) is motivated by their potency and tolerability. Both drugs are considered as preferred drugs for first-line treatment in international treatment guidelines. An understanding of the virological and immunological effects of these two combinations will provide essential information on the most appropriate regimen for use in patients who present with advanced HIV disease.

# 3. STUDY DESIGN

This is a multicentre, parallel group, randomised, open-label, non-inferiority study. At baseline, patients with no genotypic mutations for the study drugs and with a negative HLAB57 or 5701 result will be randomized to one of the 2 arms:

**Arm A: abacavir/lamivudine (Kivexa®) 1 tablet per day + raltegravir (Isentress®) 1 tablet twice a day**

**Arm B: abacavir/lamivudine (Kivexa®) 1 tablet per day + darunavir (Prezista®) 400 mg 2 tablets once a day + norvir (Ritonavir®) 1 tablet once a day**

# 4. OBJECTIVES

**Primary Endpoint:**

- Proportion of patients with undetectable viremia (HIV-1 RNA<50 copies/mL) after 48 weeks

**Secondary Endpoints(s):**

- Change in CD4+ cell count from baseline through week 48
- Time to virological rebound, defined as plasma HIV RNA >50 copies/mL measured on two consecutive occasions at least one month apart.

**Safety endpoints:**

- Incidence of adverse events (AEs)
- Incidence of serious adverse events (SAEs)
- Discontinuations due to adverse events
- Incidence of grade 3 or 4 laboratory abnormalities.

# 5. OUTCOMES

The proportion of patients attaining an HIV RNA level <50 copies/mL after 48 weeks will be the primary outcome.

# 6. TARGET POPULATION

## 6.1 Inclusion criteria

1. Males or females (inpatients or outpatients) aged 18-64 years who are HIV-1 antibody seropositive, with a CD4 count <200 cells/uL.

2. All patients should be antiretroviral-naive

3. All patients should be HLA B57 or HLA B5701 negative

4. Patients must have an HIV RNA level <500,000 copies/mL

5. Patients with an active opportunistic infection could be enrolled as long as this was diagnosed more than 2 weeks prior to screening.

6. Patients must meet the following laboratory criteria.

Neutrophil count > 1,000 cells/mm^3^

Haemoglobin > 9.0 grams/dl (men and women)

Platelet count ≥ 75,000 cells/mm^3^

Alkaline phosphatase < 3.0 the upper limit of normal

ALT and AST < 3.9 times the upper limit of normal

Total bilirubin < 1.5 times the upper limit of normal.

7. Female patients of childbearing potential must be willing to use a reliable form of contraception, which will include a medically approved form of barrier contraception.

8. Patients must be able to provide written consent to comply with study requirements.

## 6.2 Exclusion Criteria

1. Patients with genotypic mutations for any of the study drugs.

2. Patients with an opportunistic infection diagnosed in the 2 weeks prior to screening.

3. Female patients who are pregnant or breastfeeding.

4. Patients who are receiving any investigational drug or anti-neoplastic radiotherapy/chemotherapy other than local skin radiotherapy within 12 weeks before randomization.

5. Patients with a current history of intravenous drug abuse, alcohol or substance abuse.

## 6.3 Number of subjects

The planned sample size for this trial is 350 patients. Assuming an underlying response rate of 80% in each of the arms at week 48, this sample size (175 patients per arm) will provide 80% power to demonstrate non-inferiority of the raltegravir arm compared to the darunavir arm (alpha=0.025) with a non-inferiority margin of 12% [2, 9].

## 6.4 Removal of subjects from study

Subjects may be withdrawn from the study for the following reasons:

- At their own request or at the request of their legally acceptable representative
- If, in the investigator’s opinion, continuation in the study would be detrimental to the subject’s well-being
- Pregnancy
- The development of a severe reaction to any of the drugs

In all cases, the reason for withdrawal must be recorded in the case report form and the subject’s medical records.

# 7. TREATMENT REGIMENS

The study population will consist of 350 antiretroviral-naïve subjects presenting with advanced HIV disease.

At baseline, patients will be randomized 1:1 to abacavir/lamivudine (Kivexa®) 1 tablet per day + raltegravir (Isentress®) 1 tablet twice a day (Arm A) or to abacavir/lamivudine (Kivexa®) 1 tablet per day + darunavir (Prezista®) 400 mg 2 tablets once a day + norvir (Ritonavir®) 1 tablet once a day (Arm B).

Kivexa, Isentress and Prezista will be supplied by the respective pharmaceutical companies.

After 48 weeks of study treatment patients will continue the same antiretroviral regimen or will change it in accordance with the decision of the their physicians.

Switching treatments prior to the 48-week timepoint ,due to toxicity, will be permitted.

## 7.1 Prior and concomitant therapy

All medications given during the study treatment period, and/or in conjunction with a recordable adverse event must be listed in the patient’s case report form.

Drugs known to interfere with the metabolism of the study drugs are not permitted.

## 7.2 **Discontinuation**

If a patient decides not to continue with the study their treatment will be unaffected by this decision. Patients who develop a severe reaction to any of the drugs will just switch treatments and continue the study.

# 8. VISIT SCHEDULE

Patients will be followed-up at screening, baseline, week 4, week 12, week 24, week 36, week 48 and 4 weeks after the end of the treatment. At the same timepoints patients will undergo clinical laboratory testing (see paragraph 8.2).

After 48 weeks of study treatment patients will be followed according to the local clinical practice.

## 8.1 Physical examination

General physical examination will include assessment of physical status and vital signs. Blood pressure and pulse rate will be measured after the patients have been sitting comfortably for at least five minutes.

Blood pressure and pulse rate will be considered within the normal range when between 100/60-130/90 and 50-95, respectively.

## 8.2 Clinical Laboratory Tests

Haematology: Hb, RBC, haematocrit, MCV, WBC (including differentials) and platelet count.

Clinical chemistry (serum/plasma): total and unconjugated bilirubin, total protein, albumin, AST, ALT, GGT, AP, LDH, urea, creatinine, total cholesterol, HDL and LDL, triglycerides, serum amylase.

Virologic tests: HIV-RNA.

Immunologic tests: CD4 cell count (absolute number and percentage), CD8 cell count (absolute number and percentage).

All patients experiencing virological failure (HIV RNA>50 copies/mL measured on 2 consecutive occasions at least one month apart) will undergo genotypic restistance testing.

Genotyipic resistance tests used in this trial will be the same performed in accordance to local or centre clinical practice (i.e. starting treatment and in any confirmed virological rebound).

## 8.3 Pregnancy test

All female subjects will have a routine urine pregnancy test performed at screening and whenever indicated.

## 8.4 Data collection

The following data will be collected at randomization:

- Sex
- Age
- BMI (Body Mass Index)
- Risk factor for HIV
- CDC stage
- Time on antiretrovials
- Time on current antiretrovials
- Drugs in ongoing antiretroviral regimen
- Previous antiretroviral drugs
- Time below detection limit for HIV RNA

## 8.5 Adherence and Neurocognitive Evaluation

Each patient’s adherence to antiretroviral therapy will be determined by study staff and reported in the patient’s case report form at each follow-up visit

Adherence will also be measured at each visit using a visual analogue scale (Appendix 1).

All patients will undergo a routine evaluation (at each visit) of the HIV associated neurocognitive disorders by using 2 screening test: International HIV Dementia Scale and Mini Mental State Examinationa. Patients will perform neuropsychological evaluations. (Appendix 2)

# 9. STATISTICAL ANALYSIS

The primary outcome will be the proportion of patients with undetectable viremia after 48 weeks, regardless of any treatment changes.  Thus, patients who change treatments prior to the 48-week timepoint will not be considered as failures in the analysis as long as their viral load is <50 copies/ml at 48 weeks. Since we will allow switches to any drug (we are interested in the initial strategy), these do not count as failures. However, as a sensitive analysis, we should also run the analyses on an ITT population, in which we can take non-completer=failure.

  As this is a non-inferiority trial, we will calculate the difference in the proportions of patients experiencing the primary outcome in the two treatment arms and will calculate a 95% confidence interval for this.  Non-inferiority of the raltegravir arm will be demonstrated if the lower limit of the 95% confidence interval is greater than -12%. In case non-inferiority will be met, analyses for superiority will be performed.

The baseline characteristics of patients in the two treatment arms will be described using proportions (for categorical variables) and medians/means (depending on the distribution of the variables) and ranges/standard deviations (for continuous variables) as appropriate.  The mean change in CD4 cell count from baseline through week 48 will be calculated for each treatment group; changes will be compared using an unpaired t-test (assuming the differences are Normally distributed, the Mann-Whitney U test will be used if this is not the case).  Time to virological rebound will be described using the Kaplan-Meier method, with patient follow-up right-censored at the time of trial withdrawal should this occur prior to virological rebound.  Each of the safety endpoints will be summarised using proportions and will be compared across treatment groups using Chi-squared tests.

# 10. ADVERSE EVENTS (AEs) AND SERIOUS ADVERSE EVENTS (SAEs)

The investigator is responsible for reporting and documenting events falling within the protocol definitions of AEs or SAEs. During the treatment period, the investigator or designated sub-investigator shall be responsible for reporting AEs and SAEs as described in this section of the protocol. In order to satisfy international safety requirements, the investigator must include in his/her evaluation every SAE caused by participation in the study.

## 10.1 Definition of an adverse event (AE)

Per GCP, an adverse event is any untoward medical occurrence in a patient or clinical investigation subject administered a pharmaceutical product and which does not necessarily have a causal relationship with this treatment. An adverse event (AE) can therefore be any unfavourable and unintended sign (including an abnormal laboratory finding), symptom, or disease temporally associated with the use of a medicinal (investigational) product, whether or not related to the medicinal (investigational) product.

## 10.2 Assessment of Adverse Event Severity and Relationship to Treatment

An adverse event is defined as any untoward medical occurrence in a patient or clinical investigation subject, temporally associated with the use of a medicinal product, whether or not considered related to the medicinal product.

The Division of AIDS (DAIDS) in the United States has developed a graduated scale to evaluate the severity of Adverse Events and laboratory abnormalities of clinical significance during the use of antiretroviral agents. The investigator must utilize these definitions whenever possible. For abnormalities not reported on the Toxicity scale please refer to the scale reported on appendix 1 which defines the grade of severity of the event.

- **Grade 1 (Mild):** Symptoms causing no or minimal interference with usual social and functional activities
- **Grade 2 (Moderate):** Symptoms causing greater than minimal interference with usual social and functional activities
- **Grade 3 (Severe):** Symptoms causing inability to perform usual social and functional activities
- **Grade 4 (Life threatening):** Symptoms causing inability to perform basic self-care functions OR Medical or operative intervention indicated to prevent permanent impairment, persistent disability, or death

## 10.3 Definition of a serious adverse event (SAE)

Per GCP a Serious Adverse Event is any untoward medical occurrence that at any dose:

- results in death,
- is life-threatening
- requires inpatient hospitalization or prolongation of existing hospitalization
- results in persistent or significant disability/incapacity, or is a congenital anomaly/birth defect

Additionally, important medical events that may not results in death, be lifethreatening, or require hospitalization may be considered a serious adverse event when, based upon appropriate medical judgment, the may jeopardize the subject and may require medical or surgical intervention to prevent one of the outcomes listed in this definition. Example of such medical events include allergic bronchospasm requiring intensive treatment in an emergency room or at home, blood dyscrasias or convulsions that do not result in in-patient hospitalization, or the development of drug dependency or drug abuse. SAEs that occur at any time after the inclusion of the subject in the trial up to 30 days after the subject completed or discontinued the trial must be reported. In the specific circumstance of screening failures, SAEs must be collected from the time of consent signing until the subject is considered a screen failure.

## 10.4 Abnormal laboratory parameters and other abnormal values as AEs or SAEs

Abnormal laboratory values (e.g. biochemistry, haematology, urine test) or other abnormal clinical examinations (e.g. ECG, radiological examinations, vital signs) that are judged by the investigator to be clinically significant must be considered AEs or SAEs if they satisfy the respective definition criteria.

Clinically significant abnormal laboratory values or other signs diagnosed after the administration of the study drug, or which were already present at the baseline visit and worsened after the start of the study, are considered AEs or SAEs. The investigator is obliged to exercise his/her medical and scientific judgement in deciding whether the abnormal laboratory values or other abnormal clinical examinations are or are not clinically significant.

## 10.5 Reporting AEs and SAEs

Each AE and SAE that occurs during the study must be documented in the medical records of the patient in accordance with the standard clinical practice of the investigator, and on the Adverse Events/SAE page of the CRF. If there is any change in the information over time, an updated SAE report must be sent

**Follow-up of AEs or SAEs**

All AEs and SAEs must be followed up:

- until their complete resolution
- until their stabilisation
- until the event can be attributed a new aetiology
- until the patient ceases to be in the care of the Centre

## 10.8 Deadlines for SAE notification

All SAEs, whether or not deemed drug-related or expected, must be reported by the investigator or qualified designee to Promoter (Principal Investigator of Coordinating Centre) within 1 working day of first becoming aware of the event.

All SAEs should be also reported to local Ethic Committee.

## 10.9 **Suspected Unexpected Serious Adverse Reactions (SUSARs)**

The Investigator has the responsibility to report Suspected Unexpected Serious Adverse Reactions (SUSARs), that occur during the trial, to the Italian Drug Agency (AIFA), to local Ethic Committees and to the holder of the Marketing, according to the provisions of Articles. 16 (paragraphs 1 and 2) and 17 (paragraphs 3 and 5) of D. Decree 211/03 and art. 3 of the Health Ministerial Decree 17.12.04.

# 11. ETHICAL and REGULATORY ASPECT

## 11.1 Regulatory Authority Approval

In accordance with the regulations in force, the Principal Investigator must obtain approval from the appropriate Regulatory Authority before starting the clinical study. The trial will be conducted in accordance with the rules of Good Clinical Practice (GCP), the International Conference on Harmonization (ICH) and all applicable laws, including the Declaration of Helsinki of June 1964, as amended by the 59nd *World Medical Association (Seoul),* in October 2008.

## 11.2 Ethical Approval

Prior to initiation of the trial at any site, the trial, including the protocol, informed consent, and other trial documents must be approved by an appropriate Institutional Review Board (IRB) or Independent Ethics Committee (IEC). The IRB/IEC must be constituted according to applicable regulatory requirements. The investigator must guarantee that the protocol has been seen and approved by the or before starting the study. As appropriate, amendments to the protocol must also be approved by the IRB/IEC before implementation at the site. The IRB/IEC approval should be obtained in writing, clearly identifying the trial, the document reviewed (including informed consent), and the date of the review. Until such time, it will be necessary to refer to the previous version of the already approved document.

## 11.3 Informed Consent Form

The investigator or other designated personnel have the task of informing the subjects about all of the aspects and procedures of the study. The process of obtaining informed consent must comply with the regulatory procedures in force. The investigator (or a designated collaborator) and the subject must date and sign the informed consent form before the patient starts any procedure relating to the study. The subject will receive a copy of the ICF dated and signed by both parties; the original copy will be kept in the study archives. Neither the investigator nor the designated personnel should in any way exercise any coercion or influence on a subject to induce him/her to participate or continue to participate in the study. The decision of a subject to participate in the study must be completely voluntary. The investigator and designated personnel must emphasise to the subject that he/she can withdraw his/her consent at any time without any penalty or loss of any benefits to which he/she may be entitled. The written or oral information concerning the study, including the written consent form, must not contain any language that forces the subject to renounce (even only apparently) his/her legal rights, or which would exonerate the investigator, institution or sponsor from liability for negligence.

## 11.4 Duties of Investigator

In accordance with the applicable local regulations, the investigator shall make periodic reports concerning the progress of the study in his/her centre to the IEC/IRB, and notify the same at study closure. The periodic reports and closure notification are part of the responsibilities of the investigator.

## 11.5 Trial Documents and Records Retention

Following closure of the study, the investigator or head of the medical institution (where applicable) must maintain all site study records (except for those required by local regulations to be maintained elsewhere) in a safe and secure location. The records must be easily accessible when needed (e.g., for an audit or regulatory inspection) and must be available for review in conjunction with assessment of the facility, supporting systems, and relevant site staff. Where permitted by local laws/regulations or institutional policy, some or all of the records may be maintained in a format other than hard copy (e.g., microfiche, scanned, electronic); however, caution must be exercised before such action is taken. The investigator must ensure that all reproductions are legible and are a true and accurate copy of the original. In addition, they must meet accessibility and retrieval standards, including regeneration of a hard copy, if required. The investigator must also ensure that an acceptable back-up of the reproductions exists and that there is an acceptable quality control procedure in place for creating the reproductions. The minimum retention time will meet the strictest standard applicable to a particular site, as dictated by local laws/regulations, standard operating procedures, and/or institutional requirements.

## 11.6 Monitoring Arrangements

Monitoring and auditing procedures defined by the Principal Investigator and the Co-investigator will be followed in order to comply with Good Clinical Practice (GCP) guidelines*.*

# 12. PUBBLICATION

It is intended to publish the results of this study in peer-reviewed journals. The first author and co-authors will be chosen on the basis of their scientific contribution to the study and the extent of their contribution to writing the article.

# Appendix 1:VAS scale for adherence

Surname____________________

Name______________________

Date _______________________

1. In the last week how many antiretroviral pills did you take?

None All



1. In the last week how many antiretroviral pills did you take at the prescribed hours?

None All



1. In the last month how many antiretroviral pills did you take?

None All



1. In the last month how many antiretroviral pills did you take at the prescribed hours?

None All



Thank you for your cooperation

# Appendix 2: Neurocognitive evaluation

All patients will undergo a routine evaluation (at each visit) of the HIV associated neurocognitive disorders by using 2 screening test: International HIV Dementia Scale and Mini Mental State Examinationa. Among a subgroup of patients a neuropsychological evaluation will be performed.

**TEST OF THE THREE QUESTIONS (Simioni et al, AIDS 2010)**

1. Do you ever forget things (i.e. important events, including recent ones, or appointments?)
2. Do you think be slower than usual in reasoning, in planning things, or solving problems?
3. Do you have difficulties in concentrating (i.e. in a conversation, while reading a book, watching a movie?)

🞏 Yes, often 🞏 Sometimes 🞏 Never

The test is considered positive if ≥ 1 answer is "Yes, often"

**International HIV Dementia Scale (IHDS)**

1. The IHDS may be a useful screening test to identify individuals at risk for HIV dementia.
   Consists of three subtests: timed fingertapping, timed alternating hand sequence test, and recall of four items at 2 min.

   1. The number of fingertaps of the first two fingers of the nondominant hand was measured by instructing the participant to open and close the fingers as widely and as quickly as possible over a 5 seconds period.
   2. In the alternating hand sequence test individuals were asked to perform the following movement with the non-dominant hand as quickly as possible over a 10 second periods: (i) clench the hand in a fist on a flat surface; (ii) put the hand flat on the surface with the palm down; and (iii) put the hand perpendicular to the flat surface on the side of the fifth digit. The participant would then perform the sequence correctly twice for practice before the 10 seconds subtest was performed. The number of sequences correctly performed within 10s up to a maximum number of 4 was scored.
   3. Registration was measured by reciting four words to the subject and then asking him/her to repeat them immediately. The words were repeated by the examiner until the subject could repeat all four words correctly. The subject was then asked to recall the four words after the timed fingertapping and alternating hand sequence tests were performed. The number of items recalled was scored out of 4. A half-point was assigned for each correct word recalled after prompting.
   A total score out of 12 was calculated for each participant, with each of the three subtests contributing 4 points to the total score.


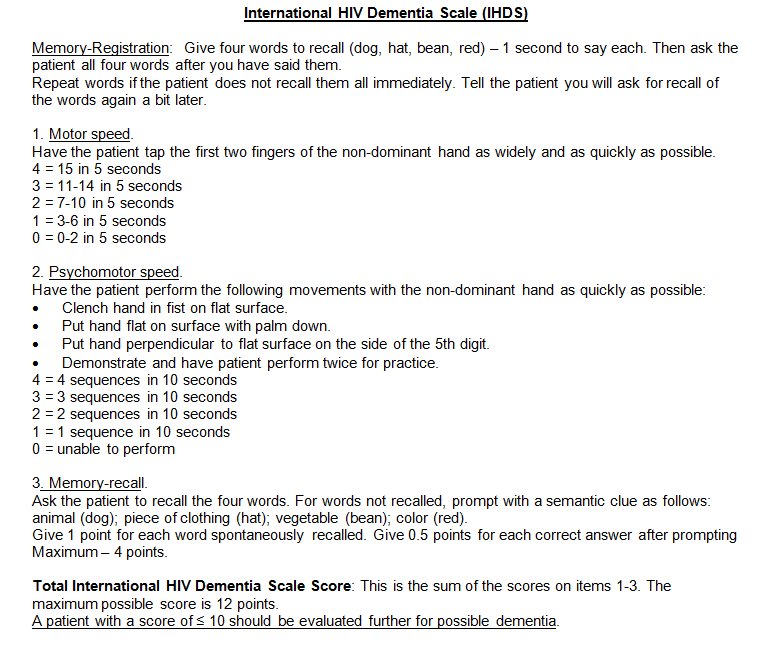


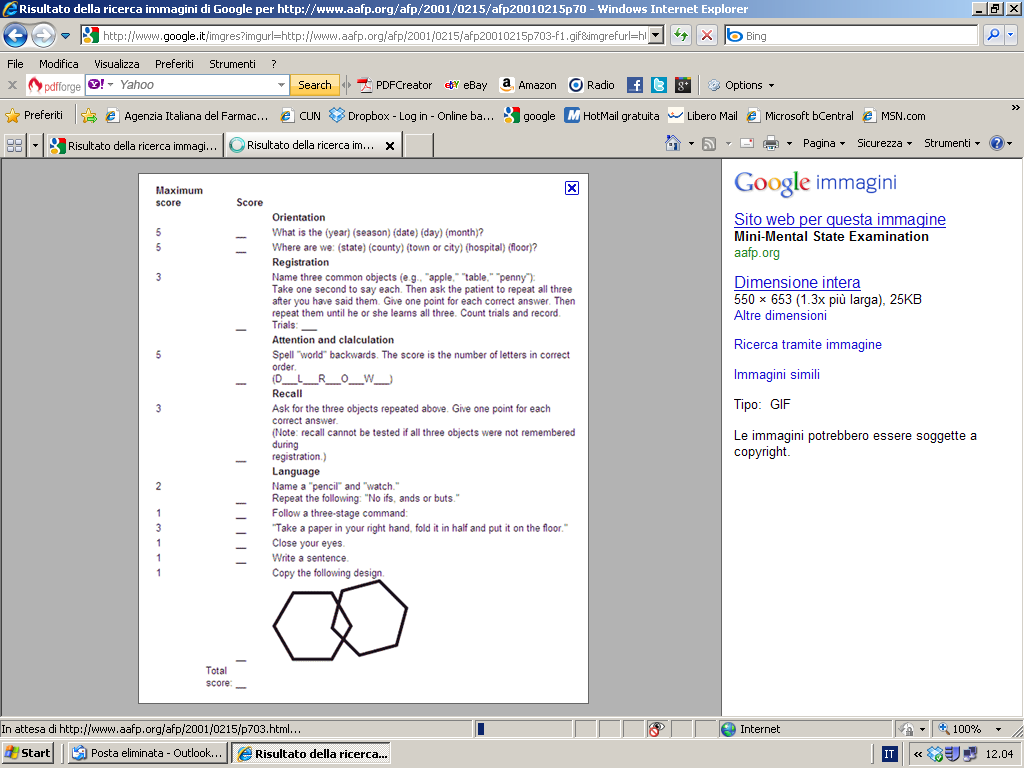


Flowchart

|  |  |  |  |  |  |  |  |  |
| --- | --- | --- | --- | --- | --- | --- | --- | --- |
| **TYPE OF VISIT** | **W -4** | **D 1** | **W 4** | **W 12** | **W 24** | **W 36** | **W 48** | **FU W 4** |
| Informed consent | X |  |  |  |  |  |  |  |
| Demographics | X |  |  |  |  |  |  |  |
| Pregnancy test | X |  |  |  |  |  |  |  |
| Incl/excl criteria | X |  |  |  |  |  |  |  |
| Anamnesis | X |  |  |  |  |  |  |  |
| Physical exam | X | X | X | X | X | X | X | X |
| Vital signs | X | X | X | X | X | X | X | X |
| Concomitant medications | X | X | X | X | X | X | X | X |
| Adverse event | X | X | X | X | X | X | X | X |
| Safety profile * | X | X | X | X | X | X | X | X |
| Neuropsychological evaluation | X | X | X | X | X | X | X | X |
| Adherence evaluation | X | X | X | X | X | X | X | X |

***** Haematology: Hb, RBC, haematocrit, MCV, WBC (including differentials) and platelet count. Clinical chemistry (serum/plasma): total and unconjugated bilirubin, total protein, albumin, AST, ALT, GGT, AP, LDH, urea, creatinine, total cholesterol, HDL and LDL, triglycerides, serum amylase. Virologic tests: HIV-RNA. Immunologic tests: CD4 cell count (absolute number and percentage), CD8 cell count (absolute number and percentage).

**DIVISION OF AIDS TABLE FOR GRADING THE SEVERITY OF**

**ADULT AND PEDIATRIC ADVERSE EVENTS**

**VERSION 1.0, DECEMBER, 2004; CLARIFICATION AUGUST 2009**

The Division of AIDS Table for Grading the Severity of Adult and Pediatric Adverse Events (“DAIDS AE Grading Table”) is a descriptive terminology which can be utilized for Adverse Event (AE) reporting. A grading (severity) scale is provided for each AE term.

This clarification of the DAIDS Table for Grading the Severity of Adult and Pediatric AE’s provides additional explanation of the DAIDS AE Grading Table and clarifies some of the parameters.

I. Instructions and Clarifications

Grading Adult and Pediatric AEs

The DAIDS AE Grading Table includes parameters for grading both Adult and Pediatric AEs. When a single set of parameters is not appropriate for grading specific types of AEs for both Adult and Pediatric populations, separate sets of parameters for Adult and/or Pediatric populations (with specified respective age ranges) are given in the Table. If there is no distinction in the Table between Adult and Pediatric values for a type of AE, then the single set of parameters listed is to be used for grading the severity of both Adult and Pediatric events of that type.

Note: In the classification of adverse events, the term “severe” is not the same as “serious.” Severity is an indication of the intensity of a specific event (as in mild, moderate, or severe chest pain). The term “serious” relates to a participant/event outcome or action criteria, usually associated with events that pose a threat to a participant’s life or functioning.

Addenda 1-3 Grading Tables for Microbicide Studies

For protocols involving topical application of products to the female genital tract, male genital area or rectum, strong consideration should be given to using Appendices I-III as the primary grading scales for these areas. The protocol would need to specifically state that one or more of the Appendices would be primary (and thus take precedence over the main Grading Table) for items that are listed in both the Appendix and the main Grading Table.

Addendum 1 - Female Genital Grading Table for Use in Microbicide Studies - [PDF](http://rcc.tech-res.com/DAIDS RCC Forms/DAIDS_AE_Grading_Table_Ver_1_0_Nov_2007_Addendum_1.pdf)

Addendum 2 - Male Genital Grading Table for Use in Microbicide Studies - [PDF](http://rcc.tech-res.com/DAIDS RCC Forms/DAIDS_AE_Grading_Table_Ver_1_0_Nov_2007_Addendum_2.pdf)

Addendum 3 - Rectal Grading Table for Use in Microbicide Studies - [PDF](http://rcc.tech-res.com/DAIDS RCC Forms/DAIDS_AE_Grading_Table_Ver_1_0_Nov_2007_Addendum_3.pdf)

Grade 5

For any AE where the outcome is death, the severity of the AE is classified as Grade 5.

Estimating Severity Grade for Parameters Not Identified in the Table

In order to grade a clinical AE that is not identified in the DAIDS AE grading table, use the category “Estimating Severity Grade” located on Page 3.

Determining Severity Grade for Parameters “Between Grades”

If the severity of a clinical AE could fall under either one of two grades (e.g., the severity of an AE could be either Grade 2 or Grade 3), select the higher of the two grades for the AE. If a laboratory value that is graded as a multiple of the ULN or LLN falls between two grades, select the higher of the two grades for the AE. For example, Grade 1 is 2.5 x ULN and Grade 2 is 2.6 x ULN for a parameter. If the lab value is 2.53 x ULN (which is between the two grades), the severity of this AE would be Grade 2, the higher of the two grades.

Values Below Grade 1

Any laboratory value that is between either the LLN or ULN and Grade 1 should not be graded.

Determining Severity Grade when Local Laboratory Normal Values Overlap with Grade 1 Ranges

In these situations, the severity grading is based on the ranges in the DAIDS AE Grading Table, even when there is a reference to the local lab LLN.

For example: Phosphate, Serum, Low, Adult and Pediatric > 14 years (Page 20) Grade 1 range is 2.50 mg/dL - < LLN. A particular laboratory’s normal range for Phosphate is 2.1 – 3.8 mg/dL. A participant’s actual lab value is 2.5. In this case, the value of 2.5 exceeds the LLN for the local lab, but will be graded as Grade 1 per DAIDS AE Grading Table.

# II. Definitions of terms used in the Table:

| Basic Self-care Functions | Adult  Activities such as bathing, dressing, toileting, transfer/movement, continence, and feeding.  Young Children  Activities that are age and culturally appropriate (e.g., feeding self with culturally appropriate eating implement). |
| --- | --- |
| LLN | Lower limit of normal |
| Medical Intervention | Use of pharmacologic or biologic agent(s) for treatment of an AE. |
| NA | Not Applicable |
| Operative Intervention | Surgical OR other invasive mechanical procedures. |
| ULN | Upper limit of normal |
| Usual Social & Functional Activities | Adult  Adaptive tasks and desirable activities, such as going to work, shopping, cooking, use of transportation, pursuing a hobby, etc.  Young Children  Activities that are age and culturally appropriate (e.g., social interactions, play activities, learning tasks, etc.). |

**Basic Self-care Functions – Adult**: Activities such as bathing, dressing, toileting, transfer/movement, continence, and feeding.

**Basic Self-care Functions – Young Children**: Activities that are age and culturally appropriate (e.g., feeding self with culturally appropriate eating implement).

**Usual Social & Functional Activities – Adult**: Adaptive tasks and desirable activities, such as going to work, shopping, cooking, use of transportation, pursuing a hobby, etc.

**Usual Social & Functional Activities – Young Children**: Activities that are age and culturally appropriate (e.g., social interactions, play activities, learning tasks, etc.).

|  | PARAMETER | | | | | | | GRADE 1 MILD | | | GRADE 2 MODERATE | | | | GRADE 3 SEVERE | | | | GRADE 4 POTENTIALLY LIFE-THREATENING | | |  |
| --- | --- | --- | --- | --- | --- | --- | --- | --- | --- | --- | --- | --- | --- | --- | --- | --- | --- | --- | --- | --- | --- | --- |
|  | ESTIMATING SEVERITY GRADE | | | | | | | | | | | | | | | | | | | | |  |
|  | Clinical adverse event NOT identified elsewhere in this DAIDS AE Grading Table | | | | | | | Symptoms causing no or minimal interference with usual social & functional activities | | | Symptoms causing greater than minimal interference with usual social & functional activities | | | | Symptoms causing inability to perform usual social & functional activities | | | | Symptoms causing inability to perform basic self-care functions OR Medical or operative intervention indicated to prevent permanent impairment, persistent disability, or death | | |  |
|  | SYSTEMIC | | | | | | | | | | | | | | | | | | | | |  |
|  | Acute systemic allergic reaction | | | | | | | Localized urticaria (wheals) with no medical intervention indicated | | | Localized urticaria with medical intervention indicated OR Mild angioedema with no medical intervention indicated | | | | Generalized urticaria OR Angioedema with medical intervention indicated OR Symptomatic mild bronchospasm | | | | Acute anaphylaxis OR Life-threatening bronchospasm OR laryngeal edema | | |  |
|  | Chills | | | | | | | Symptoms causing no or minimal interference with usual social & functional activities | | | Symptoms causing greater than minimal interference with usual social & functional activities | | | | Symptoms causing inability to perform usual social & functional activities | | | | NA | | |  |
|  | Fatigue  Malaise | | | | | | | Symptoms causing no or minimal interference with usual social & functional activities | | | Symptoms causing greater than minimal interference with usual social & functional activities | | | | Symptoms causing inability to perform usual social & functional activities | | | | Incapacitating fatigue/ malaise symptoms causing inability to perform basic self-care functions | | |  |
|  | Fever (nonaxillary) | | | | | | | 37.7 – 38.6°C | | | 38.7 – 39.3°C | | | | 39.4 – 40.5°C | | | | > 40.5°C | | |  |
|  | Pain (indicate body site)  DO NOT use for pain due to injection (See Injection Site Reactions: Injection site pain)  See also Headache, Arthralgia, and Myalgia | | | | | | | Pain causing no or minimal interference with usual social & functional activities | | | Pain causing greater than minimal interference with usual social & functional activities | | | | Pain causing inability to perform usual social & functional activities | | | | Disabling pain causing inability to perform basic self-care functions OR Hospitalization (other than emergency room visit) indicated | | |  |
|  | Unintentional weight loss | | | | | | | NA | | | 5 – 9% loss in body weight from baseline | | | | 10 – 19% loss in body weight from baseline | | | | ≥ 20% loss in body weight from baseline OR Aggressive intervention indicated [e.g., tube feeding or total parenteral nutrition (TPN)] | | |  |
|  | INFECTION | | | | | | | | | | | | | | | | | | | | |  |
|  | Infection (any other than HIV infection) | | | | | | | Localized, no systemic antimicrobial treatment indicated AND Symptoms causing no or minimal interference with usual social & functional activities | | | Systemic antimicrobial treatment indicated OR Symptoms causing greater than minimal interference with usual social & functional activities | | | | Systemic antimicrobial treatment indicated AND Symptoms causing inability to perform usual social & functional activities OR Operative intervention (other than simple incision and drainage) indicated | | | | Life-threatening consequences (e.g., septic shock) | | |  |
|  | **INJECTION SITE REACTIONS** | | | | | | | | | | | | | | | | | | | | |  |
|  | Injection site pain (pain without touching)  Or  Tenderness (pain when area is touched) | | | | | | | Pain/tenderness causing no or minimal limitation of use of limb | | | Pain/tenderness limiting use of limb OR Pain/tenderness causing greater than minimal interference with usual social & functional activities | | | | Pain/tenderness causing inability to perform usual social & functional activities | | | | Pain/tenderness causing inability to perform basic self-care function OR Hospitalization (other than emergency room visit) indicated for management of pain/tenderness | | |  |
| Injection site reaction (localized) | | | | | | | | | | | | | | | | | | | | |  | |
|  | | | **Adult > 15 years** | | | | Erythema OR Induration of 5x5 cm – 9x9 cm (or 25 cm^2^ – 81cm^2^) | | | Erythema OR Induration OR Edema  > 9 cm any diameter (or > 81 cm^2^) | | | Ulceration OR Secondary infection OR Phlebitis OR Sterile abscess OR Drainage | | | | | Necrosis (involving dermis and deeper tissue) | | |  | |
|  | | | **Pediatric ≤ 15 years** | | | | Erythema OR Induration OR Edema present but ≤ 2.5 cm diameter | | | Erythema OR Induration OR Edema > 2.5 cm diameter but < 50% surface area of the extremity segment (e.g., upper arm/thigh) | | | Erythema OR Induration OR Edema involving  ≥ 50% surface area of the extremity segment (e.g., upper arm/thigh) OR Ulceration OR Secondary infection OR Phlebitis OR Sterile abscess OR Drainage | | | | | Necrosis (involving dermis and deeper tissue) | | |  | |
|  | Pruritis associated with injection  See also Skin: Pruritis (itching - no skin lesions) | | | | | | | Itching localized to injection site AND Relieved spontaneously or with < 48 hours treatment | | | Itching beyond the injection site but not generalized OR Itching localized to injection site requiring ≥ 48 hours treatment | | | | Generalized itching causing inability to perform usual social & functional activities | | | | NA | | |  |
|  | SKIN – DERMATOLOGICAL | | | | | | | | | | | | | | | | | | | | |  |
|  | Alopecia | | | | | | | Thinning detectable by study participant (or by caregiver for young children and disabled adults) | | | Thinning or patchy hair loss detectable by health care provider | | | | Complete hair loss | | | | NA | | |  |
|  | Cutaneous reaction – rash | | | | | | | Localized macular rash | | | Diffuse macular, maculopapular, or morbilliform rash OR Target lesions | | | | Diffuse macular, maculopapular, or morbilliform rash with vesicles or limited number of bullae OR Superficial ulcerations of mucous membrane limited to one site | | | | Extensive or generalized bullous lesions OR Stevens-Johnson syndrome OR Ulceration of mucous membrane involving two or more distinct mucosal sites OR Toxic epidermal necrolysis (TEN) | | |  |
|  | Hyperpigmentation | | | | | | | Slight or localized | | | Marked or generalized | | | | NA | | | | NA | | |  |
|  | Hypopigmentation | | | | | | | Slight or localized | | | Marked or generalized | | | | NA | | | | NA | | |  |
|  | Pruritis (itching – no skin lesions)  (See also Injection Site Reactions: Pruritis associated with injection) | | | | | | | Itching causing no or minimal interference with usual social & functional activities | | | Itching causing greater than minimal interference with usual social & functional activities | | | | Itching causing inability to perform usual social & functional activities | | | | NA | | |  |
|  | CARDIOVASCULAR | | | | | | | | | | | | | | | | | | | | |  |
| Cardiac arrhythmia (general)  (By ECG or physical exam) | | | | | | | Asymptomatic AND No intervention indicated | | | Asymptomatic AND Non-urgent medical intervention indicated | | | Symptomatic, non-life-threatening AND Non-urgent medical intervention indicated | | | | | Life-threatening arrhythmia OR Urgent intervention indicated | | |  | |
|  | Cardiac-ischemia/infarction | | | | | | | NA | | | NA | | | | Symptomatic ischemia (stable angina) OR Testing consistent with ischemia | | | | Unstable angina OR Acute myocardial infarction | | |  |
|  | Hemorrhage (significant acute blood loss) | | | | | | | NA | | | Symptomatic AND No transfusion indicated | | | | Symptomatic AND Transfusion of ≤ 2 units packed RBCs (for children ≤ 10 cc/kg) indicated | | | | Life-threatening hypotension OR Transfusion of > 2 units packed RBCs (for children > 10 cc/kg) indicated | | |  |
| Hypertension | | | | | | | | | | | | | | | | | | | | |  | |
|  | | | | **Adult > 17 years**  (with repeat testing at same visit) | | | 140 – 159 mmHg systolic  OR  90 – 99 mmHg diastolic | | | 160 – 179 mmHg systolic  OR  100 – 109 mmHg diastolic | | | | **≥** 180 mmHg systolic  OR  **≥** 110 mmHg diastolic | | | | Life-threatening consequences (e.g., malignant hypertension) OR Hospitalization indicated (other than emergency room visit) | | |  | |
|  | | | | **Correction**: in Grade 2 to 160 - 179 from > 160-179 (systolic) and to ≥ 100 -109 from > 100-109 (diastolic) and  in Grade 3 to  **≥** 180 from > 180 (systolic) and to  **≥** 110 from > 110 (diastolic). | | | | | | | | | | | | | | | | |  | |
|  | | | | **Pediatric ≤ 17 years**  (with repeat testing at same visit) | | | NA | | | 91^st^ – 94^th^ percentile adjusted for age, height, and gender (systolic and/or diastolic) | | | | ≥ 95^th^ percentile adjusted for age, height, and gender (systolic and/or diastolic) | | | | Life-threatening consequences (e.g., malignant hypertension) OR Hospitalization indicated (other than emergency room visit) | | |  | |
|  | Hypotension | | | | | | | NA | | | Symptomatic, corrected with oral fluid replacement | | | | Symptomatic, IV fluids indicated | | | | Shock requiring use of vasopressors or mechanical assistance to maintain blood pressure | | |  |
|  | Pericardial effusion | | | | | | | Asymptomatic, small effusion requiring no intervention | | | Asymptomatic, moderate or larger effusion requiring no intervention | | | | Effusion with non-life threatening physiologic consequences OR Effusion with non-urgent intervention indicated | | | | Life-threatening consequences (e.g., tamponade) OR Urgent intervention indicated | | |  |
|  | Prolonged PR interval | | | | | | | | | | | | | | | | | | | | |  |
|  |  | | | | | **Adult > 16 years** | | PR interval  0.21 – 0.25 sec | | | PR interval > 0.25 sec | | | | Type II 2^nd^ degree AV block OR Ventricular pause > 3.0 sec | | | | Complete AV block | | |  |
|  |  | | | | | **Pediatric ≤ 16 years** | | 1^st^ degree AV block (PR > normal for age and rate) | | | Type I 2^nd^ degree AV block | | | | Type II 2^nd^ degree AV block | | | | Complete AV block | | |  |
| Prolonged QTc | | | | | | | | | | | | | | | | | | | | |  | |
|  | | | **Adult > 16 years** | | | | Asymptomatic, QTc interval 0.45 – 0.47 sec OR Increase interval < 0.03 sec above baseline | | | Asymptomatic, QTc interval 0.48 – 0.49 sec OR Increase in interval 0.03 – 0.05 sec above baseline | | | Asymptomatic, QTc interval ≥ 0.50 sec OR Increase in interval  ≥ 0.06 sec above baseline | | | | Life-threatening consequences, e.g. Torsade de pointes or other associated serious ventricular dysrhythmia | | | |  | |
|  | | | **Pediatric ≤ 16 years** | | | | Asymptomatic, QTc interval 0.450 –  0.464 sec | | | Asymptomatic, QTc interval 0.465 –  0.479 sec | | | Asymptomatic, QTc interval ≥ 0.480 sec | | | | Life-threatening consequences, e.g. Torsade de pointes or other associated serious ventricular dysrhythmia | | | |  | |
|  | Thrombosis/embolism | | | | | | | NA | | | Deep vein thrombosis AND No intervention indicated (e.g., anticoagulation, lysis filter, invasive procedure) | | | | Deep vein thrombosis AND Intervention indicated (e.g., anticoagulation, lysis filter, invasive procedure) | | | | Embolic event (e.g., pulmonary embolism, life-threatening thrombus) | | |  |
|  | Vasovagal episode (associated with a procedure of any kind) | | | | | | | Present without loss of consciousness | | | Present with transient loss of consciousness | | | | NA | | | | NA | | |  |
|  | Ventricular dysfunction (congestive heart failure) | | | | | | | NA | | | Asymptomatic diagnostic finding AND intervention indicated | | | | New onset with symptoms OR Worsening symptomatic congestive heart failure | | | | Life-threatening congestive heart failure | | |  |
|  | GASTROINTESTINAL | | | | | | | | | | | | | | | | | | | | |  |
|  | Anorexia | | | | | | | Loss of appetite without decreased oral intake | | | Loss of appetite associated with decreased oral intake without significant weight loss | | | | Loss of appetite associated with significant weight loss | | | | Life-threatening consequences OR Aggressive intervention indicated [e.g., tube feeding or total parenteral nutrition (TPN)] | | |  |
|  | **Comment:** Please note that, while the grading scale provided for Unintentional Weight Loss may be used as a guideline when grading anorexia, this is not a requirement and should not be used as a substitute for clinical judgment. | | | | | | | | | | | | | | | | | | | | |  |
|  | Ascites | | | | | | | Asymptomatic | | | Symptomatic AND Intervention indicated (e.g., diuretics or therapeutic paracentesis) | | | | Symptomatic despite intervention | | | | Life-threatening consequences | | |  |
|  | Cholecystitis | | | | | | | NA | | | Symptomatic AND Medical intervention indicated | | | | Radiologic, endoscopic, or operative intervention indicated | | | | Life-threatening consequences (e.g., sepsis or perforation) | | |  |
|  | Constipation | | | | | | | NA | | | Persistent constipation requiring regular use of dietary modifications, laxatives, or enemas | | | | Obstipation with manual evacuation indicated | | | | Life-threatening consequences (e.g., obstruction) | | |  |
| Diarrhea | | | | | | | | | | | | | | | | | | | | |  | |
|  | | | **Adult and Pediatric ≥ 1 year** | | | | Transient or intermittent episodes of unformed stools OR Increase of ≤ 3 stools over baseline per 24-hour period | | | Persistent episodes of unformed to watery stools OR Increase of 4 – 6 stools over baseline per 24-hour period | | | Bloody diarrhea OR Increase of ≥ 7 stools per 24-hour period OR IV fluid replacement indicated | | | | | Life-threatening consequences (e.g., hypotensive shock) | | |  | |
|  | | | **Pediatric < 1 year** | | | | Liquid stools (more unformed than usual) but usual number of stools | | | Liquid stools with increased number of stools OR Mild dehydration | | | Liquid stools with moderate dehydration | | | | | Liquid stools resulting in severe dehydration with aggressive rehydration indicated OR Hypotensive shock | | |  | |
|  | Dysphagia-Odynophagia | | | | | | | Symptomatic but able to eat usual diet | | | Symptoms causing altered dietary intake without medical intervention indicated | | | | Symptoms causing severely altered dietary intake with medical intervention indicated | | | | Life-threatening reduction in oral intake | | |  |
|  | Mucositis/stomatitis  (clinical exam)  Indicate site (e.g., larynx, oral)  See Genitourinary for Vulvovaginitis  See also Dysphagia-Odynophagia and Proctitis | | | | | | | Erythema of the mucosa | | | Patchy pseudomembranes or ulcerations | | | | Confluent pseudomembranes or ulcerations OR Mucosal bleeding with minor trauma | | | | Tissue necrosis OR Diffuse spontaneous mucosal bleeding OR Life-threatening consequences (e.g., aspiration, choking) | | |  |
|  | Nausea | | | | | | | Transient (< 24 hours) or intermittent nausea with no or minimal interference with oral intake | | | Persistent nausea resulting in decreased oral intake for 24 – 48 hours | | | | Persistent nausea resulting in minimal oral intake for > 48 hours OR Aggressive rehydration indicated (e.g., IV fluids) | | | | Life-threatening consequences (e.g., hypotensive shock) | | |  |
|  | Pancreatitis | | | | | | | NA | | | Symptomatic AND Hospitalization not indicated (other than emergency room visit) | | | | Symptomatic AND Hospitalization indicated (other than emergency room visit) | | | | Life-threatening consequences (e.g., circulatory failure, hemorrhage, sepsis) | | |  |
|  | Proctitis (functional- symptomatic)  Also see Mucositis/stomatitis  for clinical exam | | | | | | | Rectal discomfort AND No intervention indicated | | | Symptoms causing greater than minimal interference with usual social & functional activities OR Medical intervention indicated | | | | Symptoms causing inability to perform usual social & functional activities OR Operative intervention indicated | | | | Life-threatening consequences (e.g., perforation) | | |  |
|  | Vomiting | | | | | | | Transient or intermittent vomiting with no or minimal interference with oral intake | | | Frequent episodes of vomiting with no or mild dehydration | | | | Persistent vomiting resulting in orthostatic hypotension OR Aggressive rehydration indicated (e.g., IV fluids) | | | | Life-threatening consequences (e.g., hypotensive shock) | | |  |
|  | NEUROLOGIC | | | | | | | | | | | | | | | | | | | | |  |
|  | Alteration in personality-behavior or in mood (e.g., agitation, anxiety, depression, mania, psychosis) | | | | | | | Alteration causing no or minimal interference with usual social & functional activities | | | Alteration causing greater than minimal interference with usual social & functional activities | | | | Alteration causing inability to perform usual social & functional activities | | | | Behavior potentially harmful to self or others (e.g., suicidal and homicidal ideation or attempt, acute psychosis) OR Causing inability to perform basic self-care functions | | |  |
|  | Altered Mental Status  For Dementia, see Cognitive and behavioral/attentional disturbance (including dementia and attention deficit disorder) | | | | | | | Changes causing no or minimal interference with usual social & functional activities | | | Mild lethargy or somnolence causing greater than minimal interference with usual social & functional activities | | | | Confusion, memory impairment, lethargy, or somnolence causing inability to perform usual social & functional activities | | | | Delirium OR obtundation, OR coma | | |  |
|  | Ataxia | | | | | | | Asymptomatic ataxia detectable on exam OR Minimal ataxia causing no or minimal interference with usual social & functional activities | | | Symptomatic ataxia causing greater than minimal interference with usual social & functional activities | | | | Symptomatic ataxia causing inability to perform usual social & functional activities | | | | Disabling ataxia causing inability to perform basic self-care functions | | |  |
|  | Cognitive and behavioral/attentional disturbance (including dementia and attention deficit disorder) | | | | | | | Disability causing no or minimal interference with usual social & functional activities OR Specialized resources not indicated | | | Disability causing greater than minimal interference with usual social & functional activities OR Specialized resources on part-time basis indicated | | | | Disability causing inability to perform usual social & functional activities OR Specialized resources on a full-time basis indicated | | | | Disability causing inability to perform basic self-care functions OR Institutionalization indicated | | |  |
|  | CNS ischemia (acute) | | | | | | | NA | | | NA | | | | Transient ischemic attack | | | | Cerebral vascular accident (CVA, stroke) with neurological deficit | | |  |
|  | Developmental delay – **Pediatric ≤ 16 years** | | | | | | | Mild developmental delay, either motor or cognitive, as determined by comparison with a developmental screening tool appropriate for the setting | | | Moderate developmental delay, either motor or cognitive, as determined by comparison with a developmental screening tool appropriate for the setting | | | | Severe developmental delay, either motor or cognitive, as determined by comparison with a developmental screening tool appropriate for the setting | | | | Developmental regression, either motor or cognitive, as determined by comparison with a developmental screening tool appropriate for the setting | | |  |
|  | Headache | | | | | | | Symptoms causing no or minimal interference with usual social & functional activities | | | Symptoms causing greater than minimal interference with usual social & functional activities | | | | Symptoms causing inability to perform usual social & functional activities | | | | Symptoms causing inability to perform basic self-care functions OR Hospitalization indicated (other than emergency room visit) OR Headache with significant impairment of alertness or other neurologic function | | |  |
|  | Insomnia | | | | | | | NA | | | Difficulty sleeping causing greater than minimal interference with usual social & functional activities | | | | Difficulty sleeping causing inability to perform usual social & functional activities | | | | Disabling insomnia causing inability to perform basic self-care functions | | |  |
|  | Neuromuscular weakness  (including myopathy & neuropathy) | | | | | | | Asymptomatic with decreased strength on exam OR Minimal muscle weakness causing no or minimal interference with usual social & functional activities | | | Muscle weakness causing greater than minimal interference with usual social & functional activities | | | | Muscle weakness causing inability to perform usual social & functional activities | | | | Disabling muscle weakness causing inability to perform basic self-care functions OR Respiratory muscle weakness impairing ventilation | | |  |
|  | Neurosensory alteration (including paresthesia and painful neuropathy) | | | | | | | Asymptomatic with sensory alteration on exam or minimal paresthesia causing no or minimal interference with usual social & functional activities | | | Sensory alteration or paresthesia causing greater than minimal interference with usual social & functional activities | | | | Sensory alteration or paresthesia causing inability to perform usual social & functional activities | | | | Disabling sensory alteration or paresthesia causing inability to perform basic self-care functions | | |  |
|  | Seizure: (new onset)  **– Adult ≥ 18 years**  See also Seizure: (known pre-existing seizure disorder) | | | | | | | NA | | | 1 seizure | | | | 2 **–** 4 seizures | | | | Seizures of any kind which are prolonged, repetitive (e.g., status epilepticus), or difficult to control (e.g., refractory epilepsy) | | |  |
|  | Seizure: (known pre-existing seizure disorder)  **– Adult ≥ 18 years**  For worsening of existing epilepsy the grades should be based on an increase from previous level of control to any of these levels. | | | | | | | NA | | | Increased frequency of pre-existing seizures (non-repetitive) without change in seizure character OR Infrequent break-through seizures while on stable medication in a previously controlled seizure disorder | | | | Change in seizure character from baseline either in duration or quality (e.g., severity or focality) | | | | Seizures of any kind which are prolonged, repetitive (e.g., status epilepticus), or difficult to control (e.g., refractory epilepsy) | | |  |
|  | Seizure  **– Pediatric < 18 years** | | | | | | | Seizure, generalized onset with or without secondary generalization, lasting < 5 minutes with < 24 hours post ictal state | | | Seizure, generalized onset with or without secondary generalization, lasting 5 – 20 minutes with  < 24 hours post ictal state | | | | Seizure, generalized onset with or without secondary generalization, lasting  > 20 minutes | | | | Seizure, generalized onset with or without secondary generalization, requiring intubation and sedation | | |  |
|  | Syncope (not associated with a procedure) | | | | | | | NA | | | Present | | | | NA | | | | NA | | |  |
|  | Vertigo | | | | | | | Vertigo causing no or minimal interference with usual social & functional activities | | | Vertigo causing greater than minimal interference with usual social & functional activities | | | | Vertigo causing inability to perform usual social & functional activities | | | | Disabling vertigo causing inability to perform basic self-care functions | | |  |
|  | RESPIRATORY | | | | | | | | | | | | | | | | | | | | |  |
|  | Bronchospasm (acute) | | | | | | | FEV1 or peak flow reduced to  70 **–** 80% | | | FEV1 or peak flow  50 **–** 69% | | | | FEV1 or peak flow  25 **–** 49% | | | | Cyanosis OR FEV1 or peak flow < 25% OR Intubation | | |  |
| Dyspnea or respiratory distress | | | | | | | | | | | | | | | | | | | | |  | |
|  | | | **Adult ≥ 14 years** | | | | Dyspnea on exertion with no or minimal interference with usual social & functional activities | | | Dyspnea on exertion causing greater than minimal interference with usual social & functional activities | | | | Dyspnea at rest causing inability to perform usual social & functional activities | | | Respiratory failure with ventilatory support indicated | | | |  | |
|  | | | **Pediatric < 14 years** | | | | Wheezing OR minimal increase in respiratory rate for age | | | Nasal flaring OR Intercostal retractions OR Pulse oximetry 90 – 95% | | | | Dyspnea at rest causing inability to perform usual social & functional activities OR Pulse oximetry < 90% | | | Respiratory failure with ventilatory support indicated | | | |  | |
|  | MUSCULOSKELETAL | | | | | | | | | | | | | | | | | | | | |  |
|  | Arthralgia  See also Arthritis | | | | | | | Joint pain causing no or minimal interference with usual social & functional activities | | | Joint pain causing greater than minimal interference with usual social & functional activities | | | | Joint pain causing inability to perform usual social & functional activities | | | | Disabling joint pain causing inability to perform basic self-care functions | | |  |
|  | Arthritis  See also Arthralgia | | | | | | | Stiffness or joint swelling causing no or minimal interference with usual social & functional activities | | | Stiffness or joint swelling causing greater than minimal interference with usual social & functional activities | | | | Stiffness or joint swelling causing inability to perform usual social & functional activities | | | | Disabling joint stiffness or swelling causing inability to perform basic self-care functions | | |  |
|  | Bone Mineral Loss | | | | | | | | | | | | | | | | | | | | |  |
|  | |  | | | **Adult ≥ 21 years** | | | | BMD t-score  -2.5 to -1.0 | | | BMD t-score < -2.5 | | | | Pathological fracture (including loss of vertebral height) | | | | Pathologic fracture causing life-threatening consequences | | |
|  | |  | | | **Pediatric < 21 years** | | | | BMD z-score  -2.5 to -1.0 | | | BMD z-score < -2.5 | | | | Pathological fracture (including loss of vertebral height) | | | | Pathologic fracture causing life-threatening consequences | | |
|  | Myalgia (non-injection site) | | | | | | | Muscle pain causing no or minimal interference with usual social & functional activities | | | Muscle pain causing greater than minimal interference with usual social & functional activities | | | | Muscle pain causing inability to perform usual social & functional activities | | | | Disabling muscle pain causing inability to perform basic self-care functions | | |  |
|  | Osteonecrosis | | | | | | | NA | | | Asymptomatic with radiographic findings AND No operative intervention indicated | | | | Symptomatic bone pain with radiographic findings OR Operative intervention indicated | | | | Disabling bone pain with radiographic findings causing inability to perform basic self-care functions | | |  |
|  | GENITOURINARY | | | | | | | | | | | | | | | | | | | | |  |
|  | Cervicitis  (symptoms)  (For use in studies evaluating topical study agents)  For other cervicitis see Infection: Infection (any other than HIV infection) | | | | | | | Symptoms causing no or minimal interference with usual social & functional activities | | | Symptoms causing greater than minimal interference with usual social & functional activities | | | | Symptoms causing inability to perform usual social & functional activities | | | | Symptoms causing inability to perform basic self-care functions | | |  |
|  | Cervicitis  (clinical exam)  (For use in studies evaluating topical study agents)  For other cervicitis see Infection: Infection (any other than HIV infection) | | | | | | | Minimal cervical abnormalities on examination (erythema, mucopurulent discharge, or friability) OR Epithelial disruption  < 25% of total surface | | | Moderate cervical abnormalities on examination (erythema, mucopurulent discharge, or friability) OR Epithelial disruption of 25 – 49% total surface | | | | Severe cervical abnormalities on examination (erythema, mucopurulent discharge, or friability) OR Epithelial disruption  50 – 75% total surface | | | | Epithelial disruption  > 75% total surface | | |  |
|  | Inter-menstrual bleeding (IMB) | | | | | | | Spotting observed by participant OR Minimal blood observed during clinical or colposcopic examination | | | Inter-menstrual bleeding not greater in duration or amount than usual menstrual cycle | | | | Inter-menstrual bleeding greater in duration or amount than usual menstrual cycle | | | | Hemorrhage with life-threatening hypotension OR Operative intervention indicated | | |  |
|  | Urinary tract obstruction (e.g., stone) | | | | | | | NA | | | Signs or symptoms of urinary tract obstruction without hydronephrosis or renal dysfunction | | | | Signs or symptoms of urinary tract obstruction with hydronephrosis or renal dysfunction | | | | Obstruction causing life-threatening consequences | | |  |
|  | Vulvovaginitis  (symptoms)  (Use in studies evaluating topical study agents)  For other vulvovaginitis see Infection: Infection (any other than HIV infection) | | | | | | | Symptoms causing no or minimal interference with usual social & functional activities | | | Symptoms causing greater than minimal interference with usual social & functional activities | | | | Symptoms causing inability to perform usual social & functional activities | | | | Symptoms causing inability to perform basic self-care functions | | |  |
|  | Vulvovaginitis  (clinical exam)  (Use in studies evaluating topical study agents)  For other vulvovaginitis see Infection: Infection (any other than HIV infection) | | | | | | | Minimal vaginal abnormalities on examination OR Epithelial disruption  < 25% of total surface | | | Moderate vaginal abnormalities on examination OR Epithelial disruption of 25 - 49% total surface | | | | Severe vaginal abnormalities on examination OR Epithelial disruption  50 - 75% total surface | | | | Vaginal perforation OR Epithelial disruption > 75% total surface | | |  |
|  | OCULAR/VISUAL | | | | | | | | | | | | | | | | | | | | |  |
|  | Uveitis | | | | | | | Asymptomatic but detectable on exam | | | Symptomatic anterior uveitis OR Medical intervention indicated | | | | Posterior or pan-uveitis OR Operative intervention indicated | | | | Disabling visual loss in affected eye(s) | | |  |
|  | Visual changes (from baseline) | | | | | | | Visual changes causing no or minimal interference with usual social & functional activities | | | Visual changes causing greater than minimal interference with usual social & functional activities | | | | Visual changes causing inability to perform usual social & functional activities | | | | Disabling visual loss in affected eye(s) | | |  |
|  | ENDOCRINE/METABOLIC | | | | | | | | | | | | | | | | | | | | |  |
|  | Abnormal fat accumulation (e.g., back of neck, breasts, abdomen) | | | | | | | Detectable by study participant (or by caregiver for young children and disabled adults) | | | Detectable on physical exam by health care provider | | | | Disfiguring OR Obvious changes on casual visual inspection | | | | NA | | |  |
|  | Diabetes mellitus | | | | | | | NA | | | New onset without need to initiate medication OR Modification of current medications to regain glucose control | | | | New onset with initiation of medication indicated OR Diabetes uncontrolled despite treatment modification | | | | Life-threatening consequences (e.g., ketoacidosis, hyperosmolar non-ketotic coma) | | |  |
|  | Gynecomastia | | | | | | | Detectable by study participant or caregiver (for young children and disabled adults) | | | Detectable on physical exam by health care provider | | | | Disfiguring OR Obvious on casual visual inspection | | | | NA | | |  |
|  | Hyperthyroidism | | | | | | | Asymptomatic | | | Symptomatic causing greater than minimal interference with usual social & functional activities OR Thyroid suppression therapy indicated | | | | Symptoms causing inability to perform usual social & functional activities OR Uncontrolled despite treatment modification | | | | Life-threatening consequences (e.g., thyroid storm) | | |  |
|  | Hypothyroidism | | | | | | | Asymptomatic | | | Symptomatic causing greater than minimal interference with usual social & functional activities OR Thyroid replacement therapy indicated | | | | Symptoms causing inability to perform usual social & functional activities OR Uncontrolled despite treatment modification | | | | Life-threatening consequences (e.g., myxedema coma) | | |  |
|  | Lipoatrophy (e.g., fat loss from the face, extremities, buttocks) | | | | | | | Detectable by study participant (or by caregiver for young children and disabled adults) | | | Detectable on physical exam by health care provider | | | | Disfiguring OR Obvious on casual visual inspection | | | | NA | | |  |

| Laboratory | | | | | |
| --- | --- | --- | --- | --- | --- |
| PARAMETER | | GRADE 1 MILD | GRADE 2 MODERATE | GRADE 3 SEVERE | GRADE 4 POTENTIALLY LIFE-THREATENING |
| HEMATOLOGY *Standard International Units are listed in italics* | | | | | |
| Absolute CD4+ count  **–** **Adult and Pediatric  > 13 years** (HIV negative only) | | 300 – 400/mm^3^ *300 – 400/µL* | 200 – 299/mm^3^ *200 – 299/µL* | 100 – 199/mm^3^ *100 – 199/µL* | < 100/mm^3^ *< 100/µL* |
| Absolute lymphocyte count  **–** **Adult and Pediatric**  **> 13 years** (HIV negative only) | | 600 – 650/mm^3^ *0.600 x 10^9^ – 0.650 x 10^9^/L* | 500 – 599/mm^3^ *0.500 x 10^9^ –  0.599 x 10^9^/L* | 350 – 499/mm^3^ *0.350 x 10^9^ –*   *0.499 x 10^9^/L* | < 350/mm^3^ *<* *0.350 x 10^9^/L* |
| **Comment:** Values in children ≤ 13 years are not given for the two parameters above because the absolute counts are variable. | | | | | |
| Absolute neutrophil count (ANC) | | | | | |
|  | **Adult and Pediatric,  > 7 days** | 1,000 – 1,300/mm^3^ *1.000 x 10^9^ – 1.300 x 10^9^/L* | 750 – 999/mm^3^ *0.750 x 10^9^ –*  *0.999 x 10^9^/L* | 500 – 749/mm^3^ *0.500 x 10^9^ –  0.749 x 10^9^/L* | < 500/mm^3^ < *0.500 x 10^9^/L* |
|  | **Infant^*^**^†^, **2 – ≤ 7 days** | 1,250 – 1,500/mm^3^ *1.250 x 10^9^* –  *1.500 x 10^9^/L* | 1,000 – 1,249/mm^3^ *1.000 x 10^9^ –*  *1.249 x 10^9^/L* | 750 – 999/mm^3^ *0.750 x 10^9^ –  0.999 x 10^9^/L* | < 750/mm^3^ *< 0.750 x 10^9^/L* |
|  | **Infant^*^**^†^, **≤1 day** | 4,000 – 5,000/mm^3^ *4.000 x 10^9^ –  5.000 x 10^9^/L* | 3,000 – 3,999/mm^3^ *3.000 x 10^9^ –  3.999 x10^9^/L* | 1,500 – 2,999/mm^3^ *1.500 x 10^9^ –  2.999 x 10^9^/L* | < 1,500/mm^3^ *< 1.500 x 10^9^/L* |
| **Comment:**  Parameter changed from “Infant, *<* 1 day” to “Infant, **≤**1 day” | | | | | |
| Fibrinogen, decreased | | 100 – 200 mg/dL *1.00 – 2.00 g/L* OR 0.75 – 0.99 x LLN | 75 – 99 mg/dL *0.75* – *0.99 g/L* OR 0.50 – 0.74 x LLN | 50 – 74 mg/dL *0.50* – *0.74 g/L* OR 0.25 – 0.49 x LLN | < 50 mg/dL  *< 0.50 g/L* OR < 0.25 x LLN OR  Associated with gross bleeding |

| **LABORATORY** | | | | | |
| --- | --- | --- | --- | --- | --- |
| PARAMETER | | GRADE 1 MILD | GRADE 2 MODERATE | GRADE 3 SEVERE | GRADE 4 POTENTIALLY LIFE-THREATENING |
| Hemoglobin (Hgb) | | | | | |
| Comment: The Hgb values in mmol/L have changed because the conversion factor used to convert g/dL to mmol/L has been changed from 0.155 to 0.6206 (the most commonly used conversion factor).  For grading Hgb results obtained by an analytic method with a conversion factor other than 0.6206, the result must be converted to g/dL using the appropriate conversion factor for that lab. | | | | | |
|  | **Adult and Pediatric ≥ 57 days**  (HIV positive only) | 8.5 – 10.0 g/dL  *5.24 – 6.23 mmol/L* | 7.5 – 8.4 g/dL  *4.62–5.23 mmol/L* | 6.50 – 7.4 g/dL  *4.03–4.61 mmol/L* | < 6.5 g/dL  *< 4.03 mmol/L* |
|  | **Adult and Pediatric  ≥ 57 days** (HIV negative only) | 10.0 – 10.9 g/dL *6.18* – 6.79 *mmol/L*  OR Any decrease  2.5 – 3.4 g/dL *1.58 – 2.13 mmol/L* | 9.0 – 9.9 g/dL *5.55 - 6.17 mmol/L*  OR Any decrease  3.5 – 4.4 g/dL *2.14 – 2.78 mmol/L* | 7.0 – 8.9 g/dL *4.34 - 5.54 mmol/L*  OR Any decrease  ≥ 4.5 g/dL *> 2.79 mmol/L* | < 7.0 g/dL *< 4.34 mmol/L* |
| **Comment:** The decrease is a decrease from baseline | | | | | |
|  | **Infant^*^**^†^**, 36 – 56 days** (HIV positive or negative) | 8.5 – 9.4 g/dL *5.24 – 5.86 mmol/L* | 7.0 – 8.4 g/dL *4.31 – 5.23 mmol/L* | 6.0 – 6.9 g/dL *3.72 – 4.30 mmol/L* | < 6.00 g/dL *< 3.72 mmol/L* |
|  | **Infant^*^**^†^**, 22 – 35 days** (HIV positive or negative) | 9.5 – 10.5 g/dL *5.87 - 6.54 mmol/L* | 8.0 – 9.4 g/dL *4.93 – 5.86 mmol/L* | 7.0 – 7.9 g/dL *4.34 – 4.92 mmol/L* | < 7.00 g/dL *< 4.34 mmol/L* |
|  | **Infant^*^**^†^**, ≤ 21 days** (HIV positive or negative) | 12.0 – 13.0 g/dL *7.42 – 8.09 mmol/L* | 10.0 – 11.9 g/dL *6.18 – 7.41 mmol/L* | 9.0 – 9.9 g/dL *5.59- 6.17 mmol/L* | < 9.0 g/dL *< 5.59 mmol/L* |
| **Correction**: Parameter changed from “Infant *<* 21 days” to “Infant ≤ 21 days” | | | | | |
| International Normalized Ratio of prothrombin time (INR) | | 1.1 – 1.5 x ULN | 1.6 – 2.0 x ULN | 2.1 – 3.0 x ULN | > 3.0 x ULN |
| Methemoglobin | | 5.0 – 10.0% | 10.1 – 15.0% | 15.1 – 20.0% | > 20.0% |
| Prothrombin Time (PT) | | 1.1 – 1.25 x ULN | 1.26 – 1.50 x ULN | 1.51 – 3.00 x ULN | > 3.00 x ULN |
| Partial Thromboplastin Time (PTT) | | 1.1 – 1.66 x ULN | 1.67 – 2.33 x ULN | 2.34 – 3.00 x ULN | > 3.00 x ULN |
| Platelets, decreased | | 100,000 –  124,999/mm^3^ *100.000 x 10^9^ – 124.999 x 10^9^/L* | 50,000 –  99,999/mm^3^ *50.000 x 10^9^ – 99.999 x 10^9^/L* | 25,000 –  49,999/mm^3^ *25.000 x 10^9^ – 49.999 x 10^9^/L* | < 25,000/mm^3^ *< 25.000 x 10^9^/L* |
| WBC, decreased | | 2,000 – 2,500/mm^3^ *2.000 x 10^9^ –  2.500 x 10^9^/L* | 1,500 – 1,999/mm^3^ *1.500 x 10^9^ –  1.999 x 10^9^/L* | 1,000 – 1,499/mm^3^ *1.000 x 10^9^ –  1.499 x 10^9^/L* | < 1,000/mm^3^ *< 1.000 x 10^9^/L* |

| LABORATORY | | | | | |
| --- | --- | --- | --- | --- | --- |
| PARAMETER | | GRADE 1 MILD | GRADE 2 MODERATE | GRADE 3 SEVERE | GRADE 4 POTENTIALLY LIFE-THREATENING |
| CHEMISTRIES *Standard International Units are listed in italics* | | | | | |
| Acidosis | | NA | pH < normal, but ≥ 7.3 | pH < 7.3 without life-threatening consequences | pH < 7.3 with life-threatening consequences |
| Albumin, serum, low | | 3.0 g/dL – < LLN *30 g/L* – *< LLN* | 2.0 – 2.9 g/dL *20* – *29 g/L* | < 2.0 g/dL *< 20 g/L* | NA |
| Alkaline Phosphatase | | 1.25 – 2.5 x ULN^†^ | 2.6 – 5.0 x ULN^†^ | 5.1 – 10.0 x ULN^†^ | > 10.0 x ULN^†^ |
| Alkalosis | | NA | pH > normal, but ≤ 7.5 | pH > 7.5 without life-threatening consequences | pH > 7.5 with life-threatening consequences |
| ALT (SGPT) | | 1.25 – 2.5 x ULN | 2.6 – 5.0 x ULN | 5.1 – 10.0 x ULN | > 10.0 x ULN |
| AST (SGOT) | | 1.25 – 2.5 x ULN | 2.6 – 5.0 x ULN | 5.1 – 10.0 x ULN | > 10.0 x ULN |
| Bicarbonate, serum, low | | 16.0 mEq/L – < LLN *16.0 mmol/L* – *< LLN* | 11.0 – 15.9 mEq/L *11.0 – 15.9 mmol/L* | 8.0 – 10.9 mEq/L *8.0 – 10.9 mmol/L* | < 8.0 mEq/L *< 8.0 mmol/L* |
| **Comment:** Some laboratories will report this value as Bicarbonate (HCO_3_) and others as Total Carbon Dioxide (CO_2_). These are the same tests; values should be graded according to the ranges for Bicarbonate as listed above. | | | | | |
| Bilirubin (Total) | | | | | |
|  | **Adult and Pediatric > 14 days** | 1.1 – 1.5 x ULN | 1.6 – 2.5 x ULN | 2.6 – 5.0 x ULN | > 5.0 x ULN |
|  | **Infant^*^**^†^**, ≤ 14 days** (non-hemolytic) | NA | 20.0 – 25.0 mg/dL *342 – 428 µmol/L* | 25.1 – 30.0 mg/dL *429 – 513 µmol/L* | > 30.0 mg/dL *>* *513.0 µmol/L* |
|  | **Infant^*^**^†^**, ≤ 14 days** (hemolytic) | NA | NA | 20.0 – 25.0 mg/dL *342 – 428 µmol/L* | > 25.0 mg/dL > *428 µmol/L* |
| Calcium, serum, high | | | | | |
|  | **Adult and Pediatric  ≥ 7 days** | 10.6 – 11.5 mg/dL *2.65 – 2.88 mmol/L* | 11.6 – 12.5 mg/dL *2.89* – *3.13 mmol/L* | 12.6 – 13.5 mg/dL *3.14 – 3.38 mmol/L* | > 13.5 mg/dL *> 3.38 mmol/L* |
|  | **Infant^*^**^†^**, < 7 days** | 11.5 – 12.4 mg/dL *2.88* – *3.10 mmol/L* | 12.5 – 12.9 mg/dL *3.11* – *3.23 mmol/L* | 13.0 – 13.5 mg/dL *3.245 – 3.38 mmol/L* | > 13.5 mg/dL *> 3.38 mmol/L* |
| Calcium, serum, low | | | | | |
|  | **Adult and Pediatric  ≥ 7 days** | 7.8 – 8.4 mg/dL *1.95 – 2.10 mmol/L* | 7.0 – 7.7 mg/dL *1.75* – *1.94 mmol/L* | 6.1 – 6.9 mg/dL *1.53* – *1.74 mmol/L* | < 6.1 mg/dL *< 1.53 mmol/L* |
|  | **Infant^*^**^†^**, < 7 days** | 6.5 – 7.5 mg/dL *1.63 – 1.88 mmol/L* | 6.0 – 6.4 mg/dL *1.50* – *1.62 mmol/L* | 5.50 – 5.90 mg/dL *1.38* – *1.51 mmol/L* | < 5.50 mg/dL *< 1.38 mmol/L* |
|  | **Comment:** Do not adjust Calcium, serum, low or Calcium, serum, high for albumin | | | | |

| **LABORATORY** | | | | | |
| --- | --- | --- | --- | --- | --- |
| PARAMETER | | GRADE 1 MILD | GRADE 2 MODERATE | GRADE 3 SEVERE | GRADE 4 POTENTIALLY LIFE-THREATENING |
| Cardiac troponin I (cTnI) | | NA | NA | NA | Levels consistent with myocardial infarction or unstable angina as defined by the manufacturer |
| Cardiac troponin T (cTnT) | | NA | NA | NA | ≥ 0.20 ng/mL OR Levels consistent with myocardial infarction or unstable angina as defined by the manufacturer |
| Cholesterol (fasting) | | | | | |
|  | **Adult ≥ 18 years** | 200 – 239 mg/dL *5.18 – 6.19 mmol/L* | 240 – 300 mg/dL *6.20 – 7.77 mmol/L* | > 300 mg/dL > *7.77 mmol/L* | NA |
|  | **Pediatric < 18 years** | 170 – 199 mg/dL *4.40 – 5.15 mmol/L* | 200 – 300 mg/dL *5.16 – 7.77 mmol/L* | > 300 mg/dL *> 7.77 mmol/L* | NA |
| Creatine Kinase | | 3.0 – 5.9 x ULN^†^ | 6.0 – 9.9 x ULN^†^ | 10.0 – 19.9 x ULN^†^ | ≥ 20.0 x ULN^†^ |
| Creatinine | | 1.1 – 1.3 x ULN^†^ | 1.4 – 1.8 x ULN^†^ | 1.9 – 3.4 x ULN^†^ | ≥ 3.5 x ULN^†^ |

| **LABORATORY** | | | | | |
| --- | --- | --- | --- | --- | --- |
| PARAMETER | | GRADE 1 MILD | GRADE 2 MODERATE | GRADE 3 SEVERE | GRADE 4 POTENTIALLY LIFE-THREATENING |
| Glucose, serum, high | | | | | |
|  | Nonfasting | 116 – 160 mg/dL *6.44* – *8.88 mmol/L* | 161 – 250 mg/dL *8.89 – 13.88 mmol/L* | 251 – 500 mg/dL *13.89 – 27.75 mmol/L* | > 500 mg/dL *> 27.75 mmol/L* |
|  | Fasting | 110 – 125 mg/dL *6.11* – *6.94 mmol/L* | 126 – 250 mg/dL *6.95* – *13.88 mmol/L* | 251 – 500 mg/dL *13.89 – 27.75 mmol/L* | > 500 mg/dL *> 27.75 mmol/L* |
| Glucose, serum, low | | | | | |
|  | **Adult and Pediatric  ≥ 1 month** | 55 – 64 mg/dL *3.05 – 3.55 mmol/L* | 40 – 54 mg/dL *2.22 – 3.06 mmol/L* | 30 – 39 mg/dL *1.67* – *2.23 mmol/L* | < 30 mg/dL *< 1.67 mmol/L* |
|  | **Infant^*^**^†^**, < 1 month** | 50 – 54 mg/dL *2.78 – 3.00 mmol/L* | 40 – 49 mg/dL *2.22 – 2.77 mmol/L* | 30 – 39 mg/dL *1.67* – *2.21 mmol/L* | < 30 mg/dL *< 1.67 mmol/L* |
| Lactate | | ULN - < 2.0 x ULN without acidosis | ≥ 2.0 x ULN without acidosis | Increased lactate with pH < 7.3 without life-threatening consequences | Increased lactate with pH < 7.3 with life-threatening consequences |
| **Comment:** Added ULN to Grade 1 parameter | | | | | |
| LDL cholesterol (fasting) | | | | | |
|  | **Adult ≥ 18 years** | 130 – 159 mg/dL *3.37* **–** *4.12 mmol/L* | 160 – 190 mg/dL *4.13* **–** *4.90 mmol/L* | ≥ 190 mg/dL *≥ 4.91 mmol/L* | NA |
|  | **Pediatric > 2 - < 18 years** | 110 – 129 mg/dL  *2.85 – 3.34 mmol/L* | 130 – 189 mg/dL *3.35 – 4.90 mmol/L* | ≥ 190 mg/dL *≥ 4.91 mmol/L* | NA |
| Lipase | | 1.1 – 1.5 x ULN | 1.6 – 3.0 x ULN | 3.1 – 5.0 x ULN | > 5.0 x ULN |
| Magnesium, serum, low | | 1.2 – 1.4 mEq/L *0.60 – 0.70 mmol/L* | 0.9 – 1.1 mEq/L *0.45* – *0.59 mmol/L* | 0.6 – 0.8 mEq/L *0.30* – *0.44 mmol/L* | < 0.60 mEq/L *< 0.30 mmol/L* |
| Pancreatic amylase | | 1.1 – 1.5 x ULN | 1.6 – 2.0 x ULN | 2.1 – 5.0 x ULN | > 5.0 x ULN |
| Phosphate, serum, low | | | | | |
|  | **Adult and Pediatric  > 14 years** | 2.5 mg/dL – < LLN *0.81 mmol/L* – *< LLN* | 2.0 – 2.4 mg/dL *0.65* – *0.80 mmol/L* | 1.0 – 1.9 mg/dL *0.32* – *0.64 mmol/L* | < 1.00 mg/dL *< 0.32 mmol/L* |
|  | **Pediatric 1 year – 14 years** | 3.0 – 3.5 mg/dL *0.97 – 1.13 mmol/L* | 2.5 – 2.9 mg/dL *0.81* – *0.96 mmol/L* | 1.5 – 2.4 mg/dL *0.48* – *0.80 mmol/L* | < 1.50 mg/dL *< 0.48 mmol/L* |
|  | **Pediatric < 1 year** | 3.5 – 4.5 mg/dL *1.13 – 1.45 mmol/L* | 2.5 – 3.4 mg/dL *0.81* – *1.12 mmol/L* | 1.5 – 2.4 mg/dL *0.48* – *0.80 mmol/L* | < 1.50 mg/dL *< 0.48 mmol/L* |
| Potassium, serum, high | | 5.6 – 6.0 mEq/L *5.6* – *6.0 mmol/L* | 6.1 – 6.5 mEq/L *6.1* – *6.5 mmol/L* | 6.6 – 7.0 mEq/L *6.6 – 7.0 mmol/L* | > 7.0 mEq/L *> 7.0 mmol/L* |
| Potassium, serum, low | | 3.0 – 3.4 mEq/L *3.0 – 3.4 mmol/L* | 2.5 – 2.9 mEq/L *2.5* – *2.9 mmol/L* | 2.0 – 2.4 mEq/L *2.0* – *2.4 mmol/L* | < 2.0 mEq/L *< 2.0 mmol/L* |
| Sodium, serum, high | | 146 – 150 mEq/L *146* – *150 mmol/L* | 151 – 154 mEq/L *151* – *154 mmol/L* | 155 – 159 mEq/L *155* – *159 mmol/L* | ≥ 160 mEq/L *≥ 160 mmol/L* |
| Sodium, serum, low | | 130 – 135 mEq/L *130 – 135 mmol/L* | 125 – 129 mEq/L *125* – *129 mmol/L* | 121 – 124 mEq/L *121* – *124 mmol/L* | ≤ 120 mEq/L *≤ 120 mmol/L* |
| Triglycerides (fasting) | | NA | 500 – 750 mg/dL *5.65* – *8.48 mmol/L* | 751 – 1,200 mg/dL *8.49 – 13.56 mmol/L* | > 1,200 mg/dL *> 13.56 mmol/L* |

| **LABORATORY** | | | | | |
| --- | --- | --- | --- | --- | --- |
| PARAMETER | | GRADE 1 MILD | GRADE 2 MODERATE | GRADE 3 SEVERE | GRADE 4 POTENTIALLY LIFE-THREATENING |
| Uric acid | | 7.5 – 10.0 mg/dL *0.45* – *0.59 mmol/L* | 10.1 – 12.0 mg/dL *0.60* – *0.71 mmol/L* | 12.1 – 15.0 mg/dL *0.72 – 0.89 mmol/L* | > 15.0 mg/dL *> 0.89 mmol/L* |
| URINALYSIS *Standard International Units are listed in italics* | | | | | |
| Hematuria (microscopic) | | 6 – 10 RBC/HPF | > 10 RBC/HPF | Gross, with or without clots OR with RBC casts | Transfusion indicated |
| Proteinuria, random collection | | 1 + | 2 – 3 + | 4 + | NA |
| Proteinuria, 24 hour collection | | | | | |
|  | **Adult and Pediatric  ≥ 10 years** | 200 – 999 mg/24 h *0.200 – 0.999 g/d* | 1,000 – 1,999 mg/24 h *1.000 – 1.999 g/d* | 2,000 – 3,500 mg/24 h *2.000 – 3.500 g/d* | > 3,500 mg/24 h *> 3.500 g/d* |
|  | **Pediatric > 3 mo -  < 10 years** | 201 – 499 mg/m^2^/24 h *0.201 – 0.499 g/d* | 500 – 799 mg/m^2^/24 h *0.500 – 0.799 g/d* | 800 – 1,000  mg/m^2^/24 h *0.800 – 1.000 g/d* | > 1,000 mg/ m^2^/24 h *> 1.000 g/d* |

# References

1. [Late presenters in an HIV surveillance system in Italy during the period 1992-2006.](http://www.ncbi.nlm.nih.gov/pubmed/18845959) Borghi V, et al. J Acquir Immune Defic Syndr. 2008;49:282-6.
2. Prospective, Randomized, Open Label Trial of Efavirenz vs Lopinavir/Ritonavir in HIV+ Treatment-Naive Subjects With CD4+,200 cell/mm3 in Mexico. Sierra-Madero J et al. J Acquir Immune Defic Syndr 2010;53:582–588.
3. Class-Sparing Regimens for Initial Treatment of HIV-1 Infection. Riddler SA et al. for the AIDS Clinical Trials Group Study A5142 Team. N Engl J Med 2008;358:2095-106
4. Reduced bone mineral density in HIV-infected patients: prevalence and associated factors. [Cazanave C](http://www.ncbi.nlm.nih.gov/pubmed?term="Cazanave C"%5BAuthor%5D), et al. [AIDS.](javascript:AL_get(this, 'jour', 'AIDS.');) 2008;22:395-402.
5. [Abacavir-lamivudine versus tenofovir-emtricitabine for initial HIV-1 therapy.](http://www.ncbi.nlm.nih.gov/pubmed/19952143) Sax PE, et al for the AIDS Clinical Trials Group Study A5202 Team. N Engl J Med. 2009;361:2230-40.
6. [Randomized comparison of renal effects, efficacy, and safety with once-daily abacavir/lamivudine versus tenofovir/emtricitabine, administered with efavirenz, in antiretroviral-naive, HIV-1-infected adults: 48-week results from the ASSERT study.](http://www.ncbi.nlm.nih.gov/pubmed/20431394) Post FA, et al. J Acquir Immune Defic Syndr. 2010;55:49-57
7. Randomized, double-blind, placebo-matched, multicenter trial of abacavir/lamivudine or tenofovir/emtricitabine with lopinavir/ritonavir for initial HIV treatment. Smith KY, et al. AIDS 2009;23:1547-56.
8. [Virological response to initial antiretroviral regimens containing abacavir or tenofovir.](http://www.ncbi.nlm.nih.gov/pubmed/19635022) Bansi L, et al for the UK Collaborative HIV Cohort Study. J Infect Dis. 2009;200:710-4
9. [Raltegravir versus Efavirenz regimens in treatment-naive HIV-1-infected patients: 96-week efficacy, durability, subgroup, safety, and metabolic analyses.](http://www.ncbi.nlm.nih.gov/pubmed/20404738) Lennox JL, et al for the **STARTMRK** Investigators. J Acquir Immune Defic Syndr. 2010 Sep;55(1):39-48
