# Supplementary material for: A prospective randomized trial on abacavir/lamivudine plus darunavir/ritonavir or raltegravir in HIV-positive drug-naïve patients with CD4<200 cells/uL (the PRADAR study)
Source: PLoS One. 2019 Sep 27;14(9):e0222650. doi: 10.1371/journal.pone.0222650 (PMC6764686; doi:10.1371/journal.pone.0222650)
Supplement: Attachment — Submitted filename: Response to Review Comments, Cristina Mussini et al..doc [file pone.0222650.s003.doc]

**Response to Reviewers Comments**

Reviewer #1:

1. Discussion, page 18/30, line 3, following, you wrote: “Indeed, due to the low number of subjects enrolled it was not possible to perform any comparative analysis between RAL and DRV/r.” – But you have performed comparisons! And you had interesting findings, e.g. on clinical adverse events and cholesterol differences between groups. – Please clarify.

Answer: we agree with the reviewer and added a sentence in the discussion.

2. Discussion, page 19/30, first lines/first para – please consider to discuss the following facts: in NEAT001-randomized clinical trial (NCT01066962), the raltegravir-containing arm did not perform as well as the standard-of-care arm containing darunavir/ritonavir plus two nucleosidal reverse transcriptase inhibitors, in the subgroup of patients with a CD4 cell count <200/µL. Similar findings were detected in patients with another actual dual regimen in naives: NCT02831764 & NCT02831673.

Answer: we agree with the reviewer and added a paragraph in the discussion.

3. Discussion, page 19/30, lines 14 & following – see above remarks on START study findings release while PRADAR study recruitment.

Answer: we agree with the reviewer and added a sentence in the discussion.

4. Discussion, page 20/30, second para – you showed differences in week 48-outcomes of complete HIV-RNA suppression (<50 copies/mL) between arms, i.e. the primary end point (94.3 vs. 84.2%). Please consider to argue on the differences concerning the 50 copies/mL-threshold for PI vs. INSTI drug classes and to display as well the results for a 200 copies/mL-threshold, as the ACTG recommended this different limit of detection for studies on protease inhibitors.

Actually, no difference compared to <50 copies/mL was found considering a threshold of 200 copies/mL.

5. Figure 1, page 27/30: please consider to include the reasons for 7 screening failures.
they were already included in Figure 1.

Reviewer #2:

1. in Your abstract ( in the last line) you stated: the rate of virologic success is similar to that described in the literature and very far from results of the recent trials in naïve patients. this statement needs to be revisited in my view and quantified.

Answer: we agree with the reviewer and changed the last sentence since it was very vague.

2. in the last line of your introduction: >500000 should be <500000.

Answer: we have corrected the mistake, thanks.

Reviewer #3:

1. It is unfortunate that the study failed. At the same time, there is no report in the manuscript about how the study team at least tried to increase patient recruitment during study (protocol changes, increasing the number of study sites etc.)

Answer: We thank the reviewer and added a sentence in the discussion.

2. The time of recruitment is unclear. In the abstract, the authors state 3 years, in the discussion they state that they did nt expect to have such a low number of patients in 5 years.

Answer: we have corrected the mistake, thanks: the correct recruitment duration was 3 years.

Reviewer #4

1- Specify the "parametric or non-parametric tests" that were used.

The tests are already specified just one sentence after that reported by the reviewer

2- Cite the statistical software used for the analysis.

Done

3- Table 1: In addition to frequencies, provide corresponding percentages for gender, risk factors and CDC stage. For age, HIV RNA, CD4 and CD8 provide the first and third quartiles.

done
4- Provide confidence intervals for the percentage estimates in the following sentence. “According to a snapshot algorithm, on the ITT population, after 48 weeks the proportion of treatment success

was 77.3% in the RAL group and 66.7% in the DRV/r group.”

done
5- Instead of calculating the IQR, provide the first and third quartiles since they are more informative.

done
6- Provide numerical p-values rather than “NS.”

done
7- Include a table summarizing the adverse events by treatment arm. Include percentages corresponding to the frequencies.

done
